# Female sex is an independent risk factor for recurrence after ethanol Marshall bundle elimination in atrial fibrillation ablation

**DOI:** 10.3389/fcvm.2025.1556222

**Published:** 2025-04-22

**Authors:** Hongxu Chen, Xiong Xiong, Dan Chen, Xi Li, Liheng Yang, Zuowei Liu, Yanhong Chen, Jinlin Zhang

**Affiliations:** ^1^ Department of Cardiology, Wuhan Asia Heart Hospital Affiliated to Wuhan University of Science and Technology, Wuhan, Hubei, China; ^2^ Department of Medicine, School of Medicine, Wuhan University of Science and Technology, Wuhan, Hubei, China; ^3^ Department of Cardiology, Wuhan Asia General Hospital, Wuhan, Hubei, China; ^4^ Division of Cardiac Arrhythmia, Cardiac and Vascular Center, The University of Hong Kong-Shenzhen Hospital, Shenzhen, Guangdong, China

**Keywords:** atrial fibrillation, catheter ablation, vein of Marshall, sex differences, female

## Abstract

**Background:**

Atrial fibrillation (AF) exhibits gender disparities in prevalence, complications, pharmacological management, and ablation efficacy. Ethanol infusion of the vein of Marshall (EIVOM) is promising for enhancing AF ablation success rate, yet sex differences of EIVOM are lacking evidence.

**Method:**

This was a non-randomized, single-center, retrospective observational study. Patients with AF received stepwise ablations composed of EIVOM, pulmonary vein isolation, and linear ablation. The primary endpoint was defined as the recurrence of atrial tachycardia over 30 s. Propensity score matching (PSM) was performed to reduce selection bias.

**Results:**

From April 2020 to May 2022, 432 patients were included, comprising 288 male patients and 144 female patients. Compared with the male patients, the female patients were older, with worse heart function class. EIVOM success rate was significantly lower in the female patients compared with the male patients (86.1% vs. 93.4%). No significant differences in major procedural complications were observed between the male and female patients. During a median follow-up of 12 months, the female patients had significantly higher AF recurrence. Multivariate Cox regression analysis showed that female sex, body mass index <21.62, left atrial diameter >47 mm, and complex fractionated atrial electrogram ablation are independent risk factors for AF recurrence. After PSM, the AF recurrence rate remained statistically higher in the female patients compared with the male patients.

**Conclusion:**

Compared with the male patients, the female patients were older, more symptomatic, and had worse heart function. The female patients had significantly higher AF recurrence after EIVOM combined with catheter ablation.

## Introduction

Atrial fibrillation (AF) is the most common form of cardiac arrhythmia. It is associated with an increased risk of stroke, heart failure (HF), and mortality.

AF exhibited gender disparities in prevalence, complications, pharmacological management, and ablation effectiveness. Women with AF were more symptomatic and had a worse quality of life score across various measuring domains (1, 2).

Catheter ablation is an effective treatment for symptomatic patients with AF (3), however, female patients are less likely to receive catheter ablation compared with male patients (4).

It is also well known that female sex is a risk factor for AF recurrence after catheter ablation (5–8). This could be attributable to the fact that female patients who undergo ablation are older, have more comorbidities, and have a longer duration of AF (4).

Furthermore, low-voltage area (LVA) was more prevalent in female patients with AF, which is significantly associated with AF recurrence (9).

In addition to pulmonary vein isolation (PVI), the cornerstone of most ablation procedures, a promising ancillary ablation to improve the success rates of AF ablation is the ablation of Marshall bundle (MB), a structure within the left atrium (LA) that has an essential role in the initiation and maintenance of AF (10). Ethanol infusion of the vein of Marshall (EIVOM) has been shown to reduce AF recurrence rates (11–13), but the efficacy and safety of this procedure have not been extensively studied in female patients.

In this study, we aim to investigate sex-specific differences in outcomes following EIVOM in women who underwent AF ablation.

## Method

### Study design

This was a single-center, retrospective observational study conducted at a high-volume tertiary center. Patients with AF who underwent EIVOM from April 2020 to May 2022 were sequentially included. Operation exclusions included left heart or left atrial appendage thrombus formation, cancer with a predicted survival duration of less than 1 year, AF attributable to reversible etiologies, and anti-coagulation contradictions.

The study protocol was reviewed and endorsed by the Institutional Review Board of Wuhan Asia Heart Hospital. The study complied with tenets of the Helsinki Declaration and was approved by the medical ethical committee of Wuhan Asia Heart Hospital, China (No: 2018-YXKY-B017). Data were procured retrospectively and scrutinized anonymously. Informed consent was waived due to its retrospective nature by the Medical Ethical Committee of Wuhan Asia Heart Hospital, China.

In this study, the terms “sex” and “gender” are used interchangeably to refer primarily to biological differences between male and female participants, as identified at birth.

### Baseline clinical characteristics

Baseline clinical characteristics were retrospectively collected, including age, gender, body mass index (BMI), type of arrhythmia, AF symptom, New York Heart Association (NYHA) class, CHA2DS2-VASc score, HAS-BLED score, history of hypertension, diabetes mellitus, history of ischemic stroke/transient ischemic attack (TIA), coronary artery disease, history of cardiomyopathy, N-terminal B-type natriuretic peptide (NT-ProBNP), history of valve replacement, echocardiography, and medications in hospital.

### EIVOM and AF catheter ablation

For each patient, the procedure was performed under general anesthesia. EIVOM was conducted as previously described (14, 15). Briefly, the vein of Marshall (VOM) was cannulized with a JR guiding catheter, and ethanol was infused through an over-the-wire (OTW) balloon. After the VOM was cannulated, the balloon was inserted into the VOM. The ethanol injection procedure comprised three stages to produce extensive lesions. The balloon was initially positioned at the distal VOM, injecting 2–3 ml of ethanol. Deflation followed and then it was withdrawn to the mid-VOM, administering a further 2–3 ml of ethanol. Finally, the balloon was retracted to the VOM ostium, infusing an additional 2–3 ml of ethanol. Typically, 6–9 ml was instilled in the VOM. A higher dose (up to 15 ml) was employed for large multi-branching VOMs or those draining into the left atrium or left subclavian vein. The dosage infused during the EIVOM procedure was equal for male and female patients.

Antral PVI, LA roof linear ablation, and mitral isthmus (MI) linear ablation were scheduled for each patient. Tricuspid isthmus ablation, anterior LA linear ablation, or complex fractionated atrial electrograms (CFAEs) were not routinely performed. The decision to perform these ablations was based on the mechanism of the tachycardia and the electro-substrate of the left atrium.

### In-hospital management and follow-up

After the operation, the patients were transferred to the cardiac care unit (CCU) for anesthesia recovery. Trans-thoracic echocardiography (TTE) was used to rule out cardiac effusion in the CCU. Anti-coagulation therapy using warfarin [international normalized ratio (INR) of 2.0–3.0] or novel oral anticoagulants (NOACs) was prescribed for at least 3 months after the procedure. Anti-arrhythmic drugs (AADs) including amiodarone and beta-blockers were routinely administered within 3 months to reduce the recurrence of arrhythmia. Proton pump inhibitors were given for 1 month after the procedure.

Patients were followed up at 1, 3, and 12 months after the initial procedures. At the 3-month visit, all patients underwent a reassessment to evaluate their risk of stroke, determining whether it was necessary to continue their anti-coagulation therapy.

### Definition of endpoints

Peri-procedural complications, including pericardial effusion, atrial-esophageal fistula, stroke/TIA, major bleeding, and death, were collected. The primary composite endpoint was defined as follows: (1) secondary ablation within 3 months due to severe early AF recurrence symptoms; (2) any recurrence of atrial tachycardia [including AF, atrial flutter (AFL), and atrial tachycardia] over 30 s with or without oral AADs after the blanking period. AADs for AF were defined as Class I, Class II, and Class III anti-arrhythmic drugs.

Secondary ablation procedures within 3 months were considered in patients with significant quality of life decline due to AF recurrence and those who experienced severe symptoms such as palpitations, chest tightness, and dyspnea that are unmanageable by medication or electrical cardioversion. Secondary endpoints included all-cause death, stroke/TIA, and major bleeding events during follow-up.

### Statistical analysis

Statistical analyses were performed via SPSS v.29.0 statistical analysis software or R software (version 4.2.2). The methods of propensity score matching (PSM) and non-linear relationship analysis are described in the Supplementary Materials. Categorical variables were described as percentages and tested via the chi-square test. Fisher’s exact probability method and continuity correction were used for variables that did not satisfy the chi-square test conditions. The measurement data were tested for normality. Normally distributed data was presented as mean ± standard deviation (SD) (X ± s) and tested using the *t*-test. Non-normally distributed data were described using the median (interquartile range) and tested via non-parametric tests (Mann–Whitney *U*-tests). The long-term cumulative survival rates were compared via Kaplan–Meier graphs created in GraphPad Prism 9.0 software. Univariate and multivariate Cox regression analyses were used to identify predictors of AF recurrence. Both the numerical variables and the numerical variables after the maximally selected Log-rank statistic test (the cutoff value) were included in the univariate regression analysis. Variables with a *P*-value of <0.1 in the univariate analysis were included in the multivariable model. All tests were two-tailed, and *P*-values <0.05 were considered statistically significant.

## Results

### Baseline characteristic

**Figure 1:**
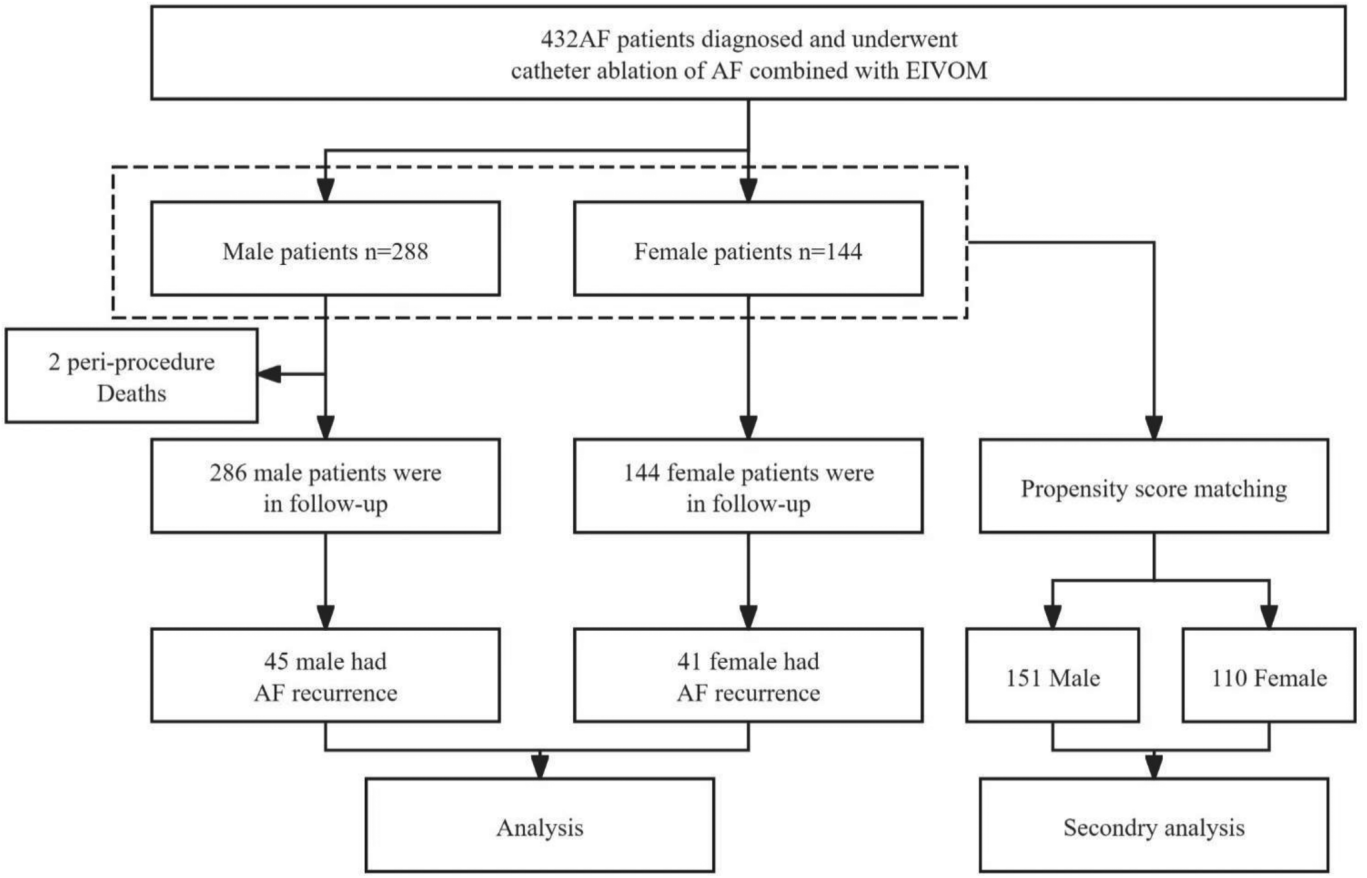
Flow chart of patients who underwent AF ablation combined with EIVOM grouped by gender. AF, atrial fibrillation; HF, heart failure; EIVOM, ethanol infusion of the vein of Marshall.

From April 2020 to May 2022, 432 patients received AF ablation combined with EIVOM, including 288 men and 144 women (Figure 1). The baseline characteristics of the male and female patients are shown in Table 1.

**Table 1:** Baseline characteristics.

Clinical characteristics	Before matching			After matching		
	Male (*n* = 288)	Female (*n* = 144)	*P*	Male (*n* = 151)	Female (*n* = 110)	*P*
Age (years)	**59.09 ± 9.95**	**64.02 ± 8.60**	<0.001**	62.68 ± 9.46	63.69 ± 8.46	0.375
BMI (kg/m^2^)	25.65 ± 3.33	25.23 ± 3.71	0.233	25.18 ± 3.21	25.04 ± 3.77	0.735
AF, *n* (%)	**272 (94.4%)**	**125 (86.8%)**	0.006**	142 (94.0%)	104 (94.5%)	0.862
AFL, *n* (%)	**16 (5.6%)**	**19 (13.2%)**	0.006**	9 (6.0%)	6 (5.5%)	0.862
Persistent AF, *n* (%)	253 (93.0%)	113 (90.4%)	0.367	134 (94.4%)	95 (91.3%)	0.356
Asymptomatic AF/AFL, *n* (%)	**56 (19.4%)**	**12 (8.3%)**	0.003**	16 (10.6%)	12 (10.9%)	0.936
Redo AF/AFL, *n* (%)	23 (8.0%)	17 (11.8%)	0.197	17 (11.3%)	7 (6.4%)	0.177
Number of previous ablations of redo AF/AFL	1 (1, 2)	1 (1, 1)	0.564	1 (1, 1)	1 (1, 1)	0.590
Time from diagnosis to first AF ablation (months)	**12 (2, 48)**	**24 (5, 60)**	0.048*	12 (3, 60)	24 (6, 60)	0.264
Time from diagnosis to first AF ablation > 12.0 months, *n* (%)	137 (47.6%)	74 (51.4%)	0.454	74 (49.0%)	54 (50.9%)	0.761
AF/AFL with HF	**120 (41.7%)**	**100 (69.4%)**	<0.001**	84 (55.6%)	72 (65.5%)	0.110
EFpHF, *n* (%)	78 (65.0%)	82 (82.0%)	—	63 (75.0%)	60 (83.4%)	—
EFrHF, *n* (%)	42 (35.0%)	18 (18.0%)	—	21 (25.0%)	12 (16.7%)	—
NYHA class						
I/II, *n* (%)	**264 (91.7%)**	**121 (84.0%)**	0.016*	137 (90.7%)	92 (83.6%)	0.125
III/IV, *n* (%)	**24 (8.3%)**	**23 (16.0%)**	0.016*	14 (9.3%)	18 (16.3%)	0.125
CHA2DS2-VASc score	**2 (1, 3)**	**3 (2, 5)**	<0.001**	**2 (1, 4)**	**3 (2, 4)**	<0.001**
HAS-BLED score	**1 (0, 1)**	**1 (0, 2)**	<0.001**	1 (0, 1)	1 (0, 2)	0.165
HAS-BLED score > 2	7 (2.4%)	6 (4.2%)	0.319	6 (4.0%)	6 (5.5%)	0.573
Hypertension, *n* (%)	**135 (46.9%)**	**91 (63.2%)**	0.001**	93 (61.6%)	72 (65.5%)	0.523
Hypertension (SBP > 160 mmHg), *n* (%)	93 (57.1%)	70 (76.9%)	0.186	69 (45.7%)	56 (50.9%)	0.405
Diabetes mellitus, *n* (%)	36 (12.5%)	16 (11.1%)	0.676	21 (13.9%)	8 (7.3%)	0.092
History of stroke/TIA, *n* (%)	30 (10.4%)	20 (13.9%)	0.288	20 (13.2%)	14 (12.7%)	0.902
Coronary artery disease, *n* (%)	63 (21.9%)	35 (24.3%)	0.570	45 (29.8%)	27 (24.5%)	0.348
History of cardiomyopathy, *n* (%)	40 (13.9%)	11 (7.6%)	0.058	23 (15.2%)	8 (7.3%)	0.077
Peripheral vascular disease, *n* (%)	14 (4.9%)	3 (2.1%)	0.197	10 (6.6%)	3 (2.7%)	0.254
Valve replacement for rheumatic heart disease, *n* (%)	**1 (0.3%)**	**13 (9.0%)**	<0.001**	1 (0.9%)	1 (0.9%)	1.000
GFR < 60, *n* (%)	**18 (6.3%)**	**19 (13.2%)**	0.015*	15 (9.9%)	16 (14.5%)	0.345
NT-ProBNP (ng/L)	**546.60 (284.55, 1,022.50)**	**840.25 (552.75, 1,511.25)**	<0.001**	775 (426.2, 1,284.75)	803 (550.2, 1,379.5)	0.424
Echocardiography						
LVEF (%)	53.15 ± 6.63	52.87 ± 6.96	0.686	53.23 ± 6.14	53.27 ± 6.44	0.957
LAD (mm)	45.04 ± 5.16	45.09 ± 4.80	0.925	45.25 ± 5.11	44.95 ± 4.71	0.640
LVEDD (mm)	**49.52 ± 4.97**	**46.84 ± 4.49**	<0.001**	47.94 ± 4.44	47.25 ± 4.22	0.158
RAD (mm)	**43.13 ± 5.53**	**41.31 ± 6.17**	0.002**	42.19 ± 5.22	42.08 ± 6.15	0.832
Medication in hospital						
Statins	122 (42.4%)	62 (43.1%)	0.891	73 (48.3%)	50 (45.5%)	0.644
ACEI/ARB/ARNI, *n* (%)	39 (13.5%)	18 (12.5%)	0.763	28 (18.5%)	12 (10.9%)	0.091
Amiodarone, *n* (%)	100 (34.7%)	53 (36.8%)	0.670	53 (35.1%)	39 (35.5%)	0.953
Beta-blockers, *n* (%)	150 (52.1%)	67 (46.5%)	0.276	79 (52.3%)	51 (46.4%)	0.342
Diltiazem, *n* (%)	17 (5.9%)	12 (8.3%)	0.341	9 (6.0%)	9 (8.2%)	0.484
DHP-CCB, *n* (%)	47 (16.3%)	25 (17.4%)	0.784	32 (21.2%)	20 (18.2%)	0.548
Diuretic, *n* (%)	**31 (10.8%)**	**26 (18.1%)**	0.035*	19 (12.6%)	20 (18.2%)	0.210
Anti-coagulation						
NOACs, *n* (%)	**282 (97.9%)**	**130 (90.3%)**	<0.001**	150 (98.0%)	109 (98.2%)	1.000
Warfarin, *n* (%)	**6 (2.1%)**	**14 (9.7%)**	<0.001**	3 (2%)	2 (1.8%)	1.000

BMI, body mass index; AF, atrial fibrillation; AFL, atrial flutter; HF, heart failure; EFpHF, heart failure with preserved left ventricular ejection fraction; EFrHF, heart failure with reduced left ventricular ejection fraction; NYHA, New York Heart Association; SBP, systolic blood pressure; TIA, transient ischemic attack; GFR, glomerular filtration rate; NT-ProBNP, N-terminal B-type natriuretic peptide; LVEF, left ventricular ejection fraction; LAD, left atrial diameter; LVEDD, left ventricular end-diastolic diameter; RAD, right atrial diameter; LVEF, left ventricular ejection fraction; ACEI, angiotensin-converting enzyme inhibitor; ARB, angiotensin receptor blocker; ARNI, angiotensin receptor neprilysin inhibitor; DHP-CCB, dihydropyridines calcium channel blocker; NOACs, novel oral anticoagulants.

Values shown are mean ± standard deviation (SD), *n* (%), or median (lower quartile, upper quartile). *P*-values were calculated by chi-squared test, *t*-test, or Mann–Whitney *U*-test, as appropriate. Statistically significant differences are indicated as *P < 0.05 and **P < 0.01. Bold values indicate statistically significant results deserving special attention.

Compared with the male patients, the female patients were older (59.09 ± 9.95 vs. 64.02 ± 8.60, *P* < 0.001), and the proportion of AFL was higher (5.6% vs. 13.2%, *P* = 0.006). The female patients were less likely to present with asymptomatic AF/AFL (54.4% vs. 78.3%, *P* < 0.001). There was no significant difference in the proportion of persistent AF between genders. Altogether, 366 (84.7%) patients had persistent AF out of 397 patients with AF, including 253 (93.0%) men and 113 (90.4%) women, and the rate of persistent AF was comparable between the sex subgroups. In terms of time from diagnosis to the first ablation, women had a longer duration [12 (2–48) vs. 24 (5–60), *P* = 0.048].

The female patients had a worse heart function class (NYHA III–IV) compared with the male patients (16% vs. 8.3%, *P* = 0.016). The female patients also exhibited a higher prevalence of HF (69.4% vs. 41.7%, *P* < 0.001) and more history of hypertension (58.2% vs. 42.2%, *P* = 0.002). They were more likely to receive diuretic therapy (18.1% vs. 10.8%, *P* = 0.035) and had higher CHA2DS2-VASc scores [3 (2–5) vs. 2 (1–3), *P* < 0.001] and higher HAS-BLED scores [1 (0–2) vs. 1 (0–1), *P* < 0.001].

Moreover, 13 (9.0%) female patients had histories of valve replacement for rheumatic heart disease compared to only 1 (0.3%) male patient (*P* < 0.001). Consequently, a higher proportion of female patients underwent warfarin anti-coagulation treatment (9.7% vs. 2.1%, *P* < 0.001). The female patients showed a higher percentage of renal impairment (13.2% vs. 6.3%, *P* = 0.015) and a higher average NT-ProBNP level than the male patients [840.25 (552.75–1,511.25) vs. 546.60 (284.55–1,022.50), *P* < 0.001].

All the patients underwent ultrasound cardiography on admission. Compared to the male patients, the female patients exhibited a larger right atrial diameter (RAD) (43.13 ± 5.53 vs. 41.31 ± 6.17, *P* = 0.002) and a greater left ventricular end-diastolic diameter (LVEDD) (49.52 ± 4.97 vs. 46.84 ± 4.49, *P* < 0.001).

After 1:1.5 PSM for age, HF, hypertension, HAS-BLED score, NT-proBNP, glomerular filtration rate (GFR) below 60, anti-coagulation, echocardiographic measurements, EIVOM success, and ablation strategy, 151 patients were included in the male group and 110 in the female group.

Baseline characteristics were then comparable between the two groups after PSM, and gender-related differences were still statistically significant for the CHA2DS2-VASc score (Table 1). A scatter plot displays the absolute standardized mean differences (SMD) of various covariates before and after PSM (Supplementary Figure S1).

### Procedure and complications

**Figure 2:**
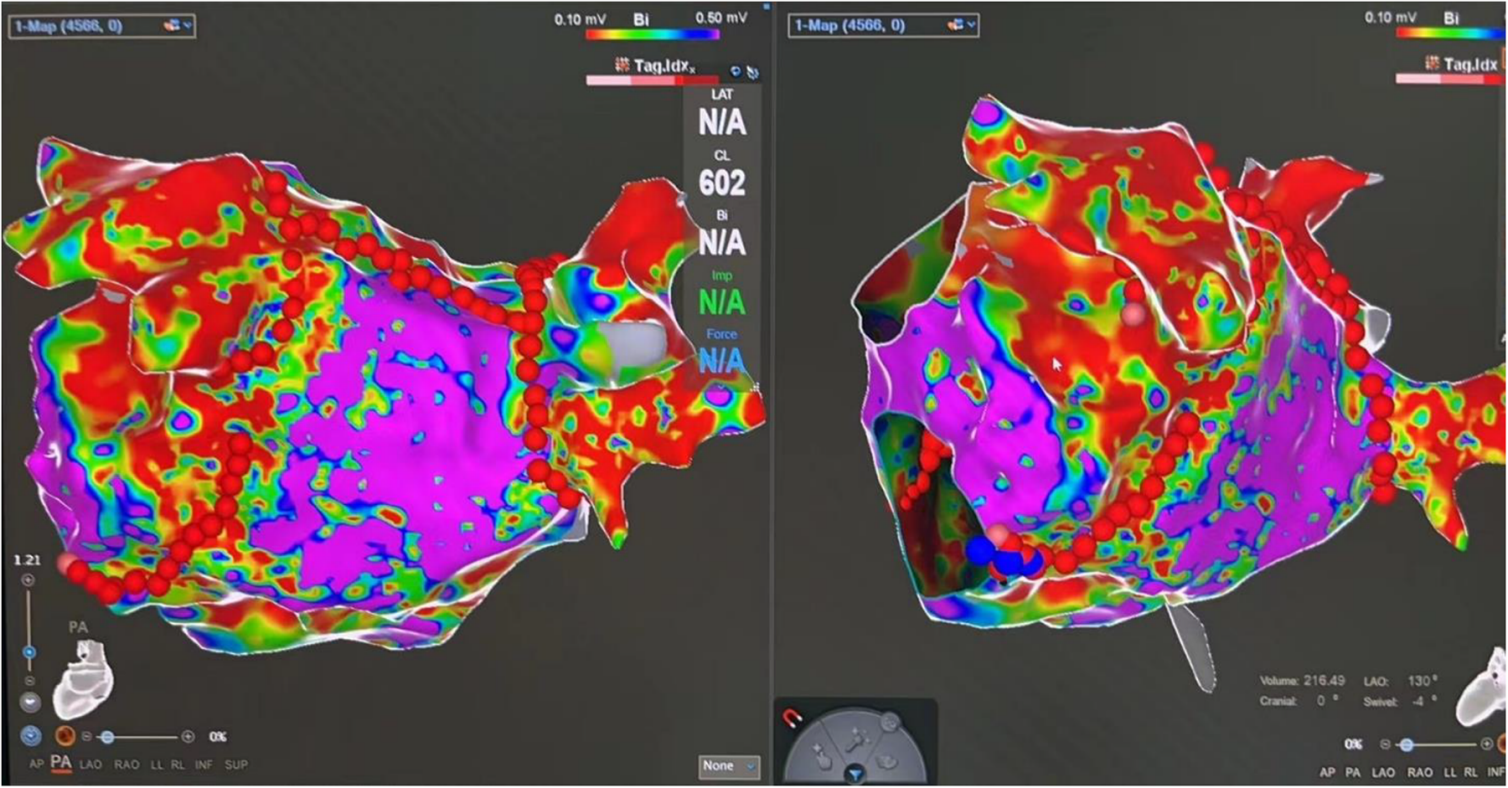
A typical left atrial electroanatomical map after pulmonary vein isolation and linear ablation. The red dots indicate the ablation sites. A low-voltage area was defined as an area with bipolar voltage between 0.1 and 0.5 mV. Extensive low-voltage areas at the mitral isthmus and left atrium ridge can be seen after ethanol infusion of the vein of Marshall.

Table 2 shows the characteristics of the procedure and peri-procedure complications. Figure 2 is a typical left atrial electroanatomical map after PVI and linear ablation. Notably, the female patients had a lower rate of VOM presence upon coronary sinus (CS) angiography (91.0% vs. 95.8%, *P* = 0.041), and they also showed a significantly lower success rate of EIVOM (93.4% vs. 86.1%, *P* = 0.013). More female patients with AF experienced conversion to AFL during ablation (9.6% vs. 19.4%, *P* = 0.007).

**Table 2:** Procedure and complications.

Procedural data	Before matching			After matching		
	Male (*n* = 288)	Female (*n* = 144)	*P*	Male (*n* = 151)	Female (*n* = 110)	*P*
Procedure						
Presence of VOM, *n* (%)	**276 (95.8%)**	**131 (91.0%)**	0.041*	141 (93.4%)	102 (92.7%)	0.838
EIVOM success, *n* (%)	**269 (93.4%)**	**124 (86.1%)**	0.013*	137 (90.7%)	97 (88.2%)	0.505
AF, *n* (%)	**272 (94.4%)**	**125 (86.8%)**	0.006**	142 (94.0%)	104 (94.5%)	0.862
AF terminates spontaneously during ablation, *n* (%)	**16 (5.9%)**	**10 (8.0%)**	**0.428**	12 (8.5%)	8 (7.7%)	0.830
AF transformed into AFL during ablation, *n* (%)	26 (9.6%)	24 (19.4%)	0.007**	12 (8.5%)	17 (16.3%)	0.058
PVI success, *n* (%)	288 (100%)	144 (100%)	1.000	151 (100%)	110 (100%)	1.000
MI ablation, *n* (%)	274 (95.1%)	135 (93.8%)	0.544	141 (57.3%)	105 (95.5%)	0.477
MI block, *n* (%)	268 (97.8%)	131 (97.0%)	0.634	138 (97.9%)	102 (97.1%)	1.000
CS ablation, *n* (%)	182 (66.4%)	102 (75.6%)	0.059	93 (66.0%)	80 (76.2%)	0.082
Roof linear ablation, *n* (%)	263 (91.3%)	124 (86.1%)	0.095	133 (88.1%)	102 (92.7%)	0.216
Anterior linear ablation, *n* (%)	**1 (0.3%)**	**11 (7.6%)**	<0.001**	1 (0.9%)	1 (0.9%)	1.000
Posterior BOX ablation, *n* (%)	**12 (4.2%)**	**17 (11.8%)**	0.003**	12 (7.9%)	9 (8.2%)	0.945
CAFE ablation, *n* (%)	**22 (7.6%)**	**21 (14.6%)**	0.023**	18 (11.9%)	14 (12.7%)	0.844
Tricuspid isthmus linear ablation, *n* (%)	26 (9.0%)	15 (10.4%)	0.642	16 (10.6%)	11 (10.0%)	0.876
Peri-procedural complications						
All pericardial effusion, *n* (%)	111 (38.5%)	66 (45.8%)	0.146	62 (41.1%)	49 (44.5%)	0.574
Thickness (mm)	7.46 ± 2.94	7.28 ± 3.60	0.720	7.88 ± 3.56	7.48 ± 4.00	0.582
Major pericardial effusion (≥10 mm), *n* (%)	15 (5.2%)	6 (4.2%)	0.635	12 (7.9%)	4 (3.6%)	0.152
Thickness (mm)	11 (10, 14)	13 (10, 18)	0.677	13.25 ± 4.50	16.75 ± 9.61	0.526
All cardiac tamponade, *n* (%)	1 (0.3%)	3 (2.0%)	0.110	0 (0%)	2 (1.8%)	1.000
Cardiac tamponade during procedure, *n* (%)	1 (0.3%)	1 (0.7%)	—	0	1 (0.9%)	—
Peri-procedural cardiac tamponade, *n* (%)	0	2 (1.4%)	—	0	1 (0.9%)	—
Delayed cardiac tamponade, *n* (%)	1 (0.3%)	0	—	0	0	—
Subcutaneous hematoma, *n* (%)	1 (0.3%)	1 (0.7%)	1.000	1 (0.6%)	1 (0.9%)	1.000
Phrenic nerve injury, *n* (%)	0	1 (0.7%)	0.333	0	0	—
Stroke/TIA, *n* (%)	2 (0.7%)	0	0.555	0	0	—
Atrial-esophageal fistula, *n* (%)	2 (0.7%)	0	0.555	0	0	—
Death, *n* (%)	2 (0.7%)	0	0.555	0	0	—

VOM, vein of Marshall; EIVOM, ethanol infusion of the vein of Marshall; AF, atrial fibrillation; AFL, atrial flutter; PVI, pulmonary vein isolation; MI, mitral isthmus; CS, coronary sinus; CAFEs, complex fractionated atrial electrograms; TIA, transient ischemic attack.

Values shown are mean ± standard deviation (SD), *n* (%), or median (lower quartile, upper quartile). *P*-values were calculated by chi-squared test, *t*-test, or Mann–Whitney *U*-test, as appropriate. Statistically significant differences are indicated as *P < 0.05 and **P < 0.01. Bold values indicate statistically significant results deserving special attention.

All the patients achieved PVI success during the procedure. In total, 274 (95.1%) male patients and 135 (93.8%) female patients underwent MI linear ablation. Furthermore, 182 male patients and 102 female patients needed additional ablations within the CS to achieve MI block (*P* = 0.059). MI block rates were equally high in both groups (97.8% vs. 97.0%, *P* = 0.634). Moreover, 263 (91.3%) male patients and 124 (86.1%) female patients underwent LA roof linear ablation (*P* = 0.095). More female patients underwent linear ablation, attributable to their higher rate of anterior linear ablation (7.6% vs. 0.3%, *P* < 0.001), posterior BOX ablation (11.8% vs. 4.2%, *P* = 0.003), and CAFE ablation (14.6% vs. 7.6%, *P* = 0.023).

No significant differences in major procedural complications were observed between the male and female patients. There were two deaths due to atrial-esophageal fistula, which appears to be a high proportion for the size of the study population. However, the elevated incidence observed might be coincidental and not representative of the general trend.

After PSM, the two cases of atrial-esophageal fistula were excluded, and there were no significant differences in ablation sites and complications between the male and female patients (Table 2). A scatter plot displays the absolute SMD of various covariates before and after PSM (Supplementary Figure S1).

### Clinical outcomes during follow-up

The events during the follow-up period are described in Table 3. Male patients were followed up for 365 (365–589) days and female patients for 365 (365–650) days (*P* = 0.68). In total, 43.7% of the patients remained on AAD medication during the follow-up period. The disparity between male and female patients (40.2% vs. 50.7%, *P* = 0.039) was primarily attributed to differences in the utilization of beta-blockers (33.9% vs. 43.1%). During the follow-up, AF recurrence occurred in 86 (20.0%) patients, including 45 (15.7%) male patients and 41 (28.5%) female patients. Among the recurrences in patients without AAD, 22 (7.7%) were in the male group and 18 (12.5%) were in the female group. The sex difference was significant [hazard ratio (HR) 0.49, 95% confidence interval (CI) 0.31–0.77, *P* = 0.001]. Among the total recurrences, 69 (16.0%) patients recurred after the blanking period of 90 days, including 35 (12.2%) men and 34 (23.6%) women (HR: 0.43, 95% CI: 0.27–0.77, *P* < 0.001). There were statistically significant gender differences for both AF (HR: 0.56, 95% CI: 0.32–0.97, *P* = 0.035) and AFL recurrence (HR: 0.39, 95% CI: 0.18–0.84, *P* = 0.013).

**Table 3:** Clinical outcomes during follow-up.

Variables	Before matching				After matching		
	All (*n* = 430)	Male (*n* = 286)	Female (*n* = 144)	*P*	Male (*n* = 151)	Female (*n* = 110)	*P*
Follow-up period (days)	365 (365, 589)	365 (365, 650)	365 (365, 544)	0.680	365 (365, 704.5)	365 (365, 498.5)	0.345
Use of AAD at follow-up	**188** **(43.7%)**	**115** **(40.2%)**	**73** **(50.7%)**	0.039*	69 (45.7%)	49 (47.3%)	0.854
AAD type, *n* (%)							
Amiodarone/dronedarone	27 (6.3%)	16 (5.6%)	11 (7.6%)	—	11 (7.3%)	9 (8.2%)	—
Beta-blockers	159 (36.9%)	97 (33.9%)	62 (43.1%)	—	47 (31.1%)	40 (36.4%)	—
Propafenone	2 (0.5%)	2 (0.7%)	0	—	1 (0.7%)	0	—
Recurrence							
All recurrence, *n* (%)	**86 (20.0%)**	**45 (15.7%)**	**41 (28.5%)**	0.001**	**28 (18.5%)**	**31 (28.2%)**	0.025*
Recurrence after blanking period, *n* (%)	**69 (16.0%)**	**35 (12.2%)**	**34 (23.6%)**	<0.001**	**26 (17.2%)**	**21 (19.1%)**	0.011*
All recurrence without AAD	**40 (9.3%)**	**22 (7.7%)**	**18 (12.5%)**	0.004**	12 (7.9%)	13 (11.8%)	0.096
AF recurrence, *n* (%)	**52 (12.1%)**	**29 (10.1%)**	**23 (16.0%)**	0.035*	17 (11.3%)	18 (16.4%)	0.115
AFL recurrence, *n* (%)	**26 (6.1%)**	**12 (4.2%)**	**14 (9.7%)**	0.013*	7 (4.6%)	9 (8.1%)	0.202
AT recurrence, *n* (%)	8 (1.9%)	4 (1.4%)	4 (2.8%)	0.145	4 (2.6%)	4 (3.6%)	0.371
Stroke/TIA, *n* (%)	5 (1.2%)	4 (1.4%)	1 (0.7%)	0.317	2 (1.3%)	1 (0.9%)	0.755
All-cause death, *n* (%)	6 (1.4%)	5 (1.7%)	1 (0.7%)	0.212	4 (2.6%)	1 (0.9%)	0.345
Death for cardiac causes, *n* (%)	1 (0.2%)	1 (0.3%)	0	—	1 (0.3%)	0	—
Major bleeding, *n* (%)	4 (0.9%)	3 (1.0%)	1 (0.7%)	0.513	1 (0.7%)	1 (0.9%)	0.798
Cerebral hemorrhage, *n* (%)	1 (0.2%)	1 (0.3%)	0	—	0	0	—
Gastrointestinal hemorrhage, *n* (%)	3 (0.7%)	2 (0.7%)	1 (0.7%)	—	1 (0.7%)	1 (0.9%)	—

AAD, anti-arrhythmic drugs; AF, atrial fibrillation; AFL, atrial flutter; AT, atrial tachycardia; TIA, transient ischemic attack.

Values shown are *n* (%) or median (lower quartile, upper quartile).

Statistically significant differences are indicated as *P < 0.05 and **P < 0.01. Bold values indicate statistically significant results deserving special attention.

Stroke or TIA occurred in five (1.2%) patients, including four male patients and one female patient. All-cause death occurred in six (1.4%) patients, including one (0.3%) patient who died from cardiac causes. Major bleeding occurred in four (0.9%) patients, which included one cerebral hemorrhage and three gastrointestinal hemorrhages. No significant differences were observed between the male and female patients in terms of these events.

However, after matching, the clinical outcomes demonstrated notable differences between the male and female patients (Table 3).

Several patients received their second ablation during the blanking period. They had severe symptoms that were refractory to AADs. A detailed description of these cases is provided in the Supplementary Materials.

**Figure 3:**
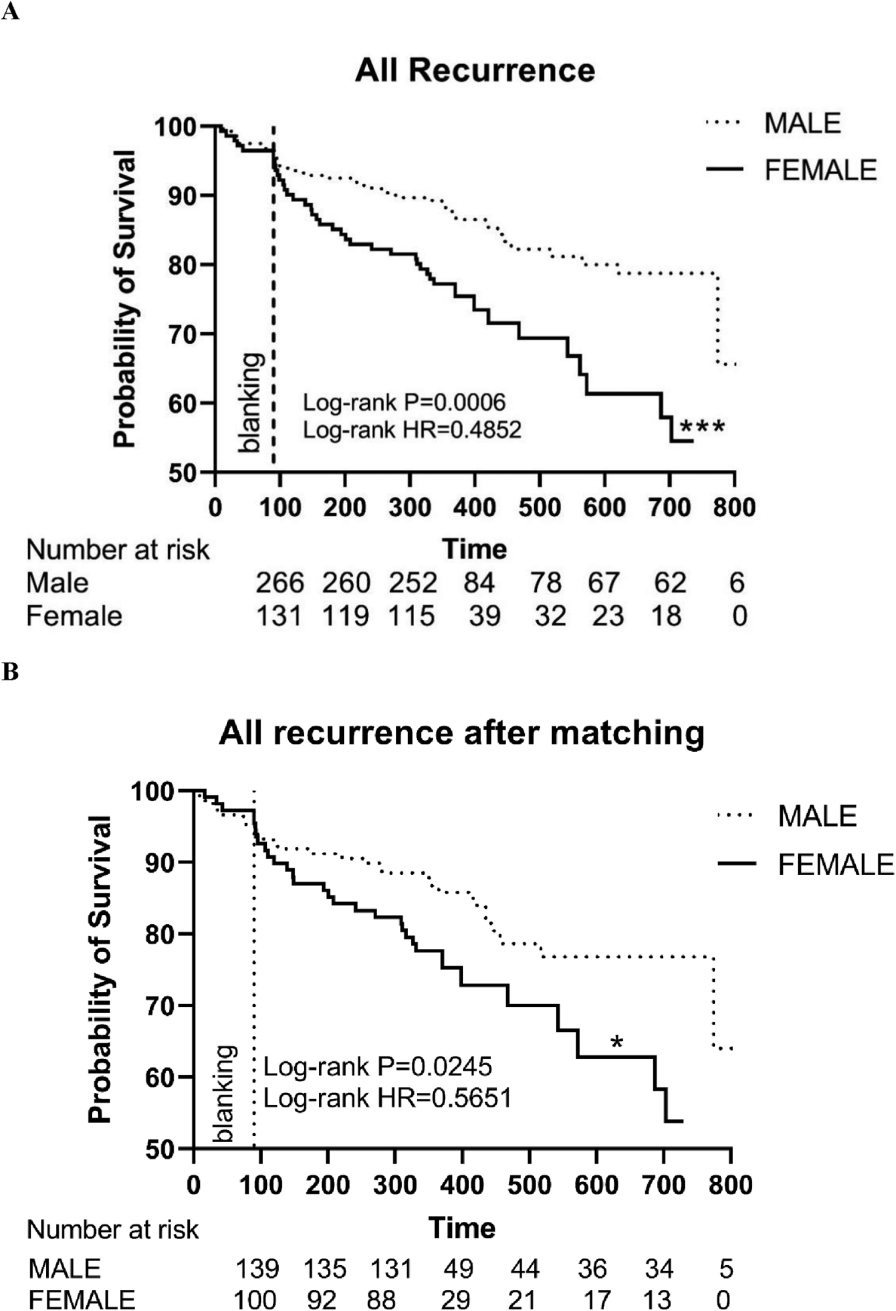
**(A)** The Kaplan–Meier graph showing cumulative survival comparison according to gender before matching; **(B)** The Kaplan–Meier graph showing cumulative survival comparison according to gender after matching. Statistically significant differences are indicated as follows: *P < 0.05; **P < 0.01; ***P < 0.001; ****P < 0.0001. HR, hazard ratio.

The Kaplan–Meier survival curve showed a significant difference in recurrence between the male and female patients (HR: 0.4852, Log-rank *P* = 0.0006) (Figure 3A).

After PSM, the Kaplan–Meier survival curve still showed a significant difference in the primary endpoints (HR: 0.5651, Log-rank *P* = 0.0245) between the male and female patients (Figure 3B).

**Figure 4:**
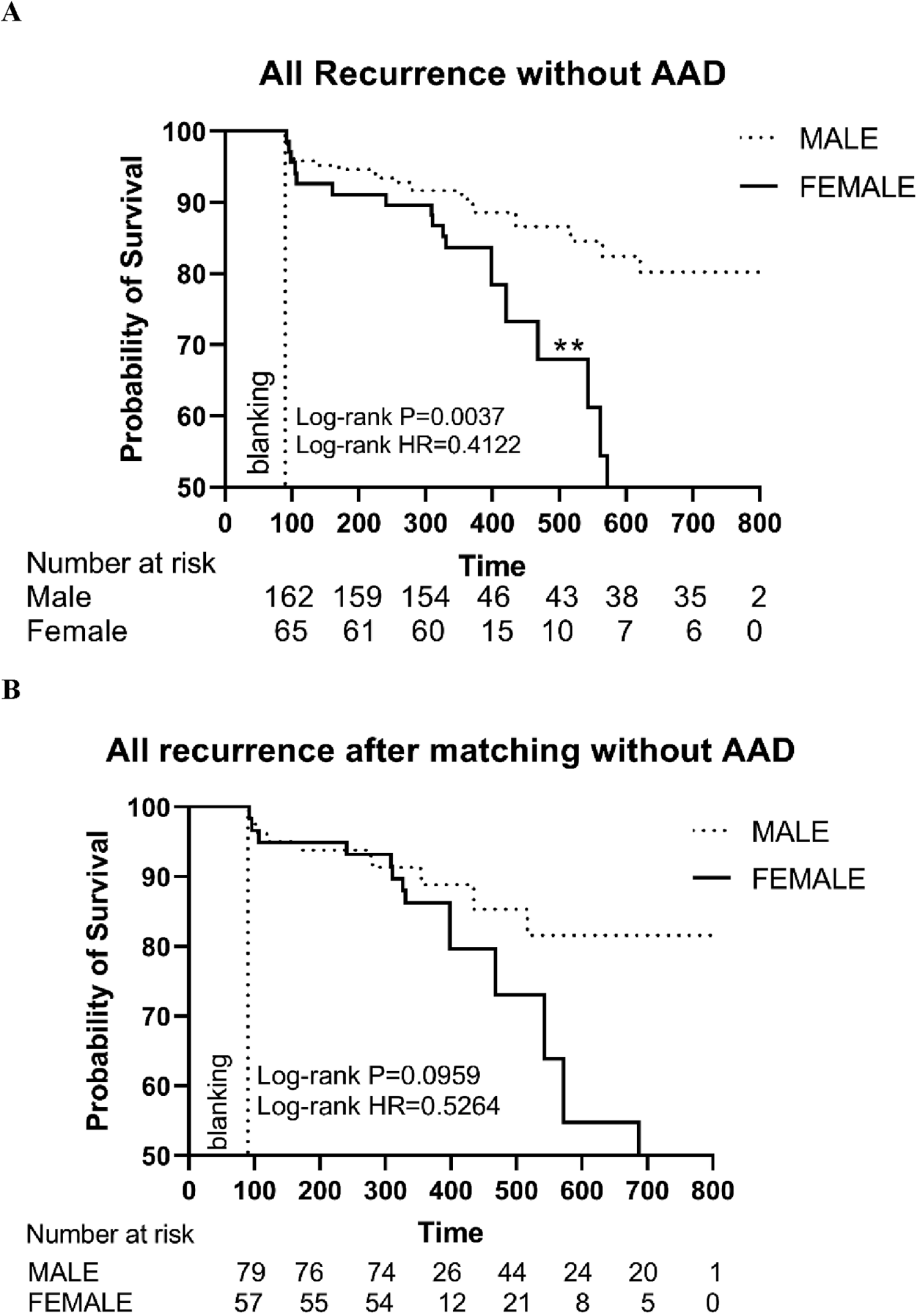
**(A)** The Kaplan–Meier graph showing cumulative survival comparison according to gender before matching; **(B)** The Kaplan–Meier graph showing cumulative survival comparison according to gender after matching. Statistically significant differences are indicated as follows: *P < 0.05; **P < 0.01; ***P < 0.001; ****P < 0.0001. HR, hazard ratio; AAD, anti-arrhythmic drugs.

Similarly, among patients without AAD, the female group showed a higher recurrence rate (HR: 0.4122, Log-rank *P* = 0.0037) (Figure 4A). After PSM, the Kaplan–Meier survival curve still showed no significant difference in recurrence without AAD (HR: 0.5264, Log-rank *P* = 0.0959) (Figure 4B).

### Survival regression analysis

In the univariate Cox regression analysis, female sex, BMI < 21.62, left atrial diameter (LAD) > 47 mm, and CAFE ablation were meaningful predictors for the primary endpoint (Table 4). Variables that were included in the univariate analysis were shown in Supplementary Table S1.

**Table 4:** Cox regression table before and after matching.

Variable	Univariate		Multivariable	
	HR (95% CI)	*P*	HR (95% CI)	*P*
(A) Before matching				
Female sex	**2.09 (1.36–3.21)**	<0.001**	**1.96 (1.27–3.02)**	0.002**
Age > 69	1.60 (0.98–2.62)	0.061		
BMI < 21.62	**2.16 (1.25–3.72)**	0.006**	**2.05 (1.18–3.56)**	0.011**
Time from diagnosis to first AF ablation >12.0 months	1.44 (0.94–2.20)	0.096		
Valve replacement	2.51 (1.09–5.76)	0.030*		
CHA2DS2-VASc score ≥ 5	2.00 (1.19–3.37)	0.009**		
LVEF < 54%	1.48 (0.97–2.27)	0.071		
LAD > 47 mm	**1.64 (1.07–2.52)**	0.025*	**1.81 (1.17–2.81)**	0.007**
LVEDD < 45 mm	1.87 (1.15–3.04)	0.012*		
RAD > 40 mm	1.48 (0.94–2.31)	0.088		
Posterior BOX ablation	2.16 (1.08–4.33)	0.030*		
CAFE ablation	**2.39 (1.38–4.12)**	0.002**	**2.11 (1.22–3.66)**	0.008**
(B) After matching				
Female gender	**2.07 (1.19–3.60)**	0.010*	**2.20 (1.23–3.76)**	0.007**
BMI < 21.62	1.86 (0.93–3.72)	0.080		
Valve replacement	4.68 (1.86–11.82)	0.001**		
HAS-BLED score ≥ 2	0.48 (0.21–1.09)	0.078		
LAD > 47 mm	**1.69 (0.96–2.96)**	**0.068**	**1.79 (1.02–3.14)**	0.044*
Posterior BOX ablation	2.53 **(**1.07–5.98)	0.035*		
CAFE ablation	1.89 (0.95–3.78)	0.072		

HR, hazard ratio; CI, confidence interval; BMI, body mass index; LVEF, left ventricular ejection fraction; LAD, left atrial diameter; LVEDD, left ventricular end-diastolic diameter; RAD, right atrial diameter; LVEF, left ventricular ejection fraction; EIVOM, ethanol infusion of the vein of Marshall; CAFE, complex fractionated atrial electrogram.

Statistically significant differences are indicated as *P < 0.05 and **P < 0.01. Variables that were not statistically significant in the univariate analysis are shown in Supplementary Materials.

Bold values indicate statistically significant results deserving special attention.

**Figure 5:**
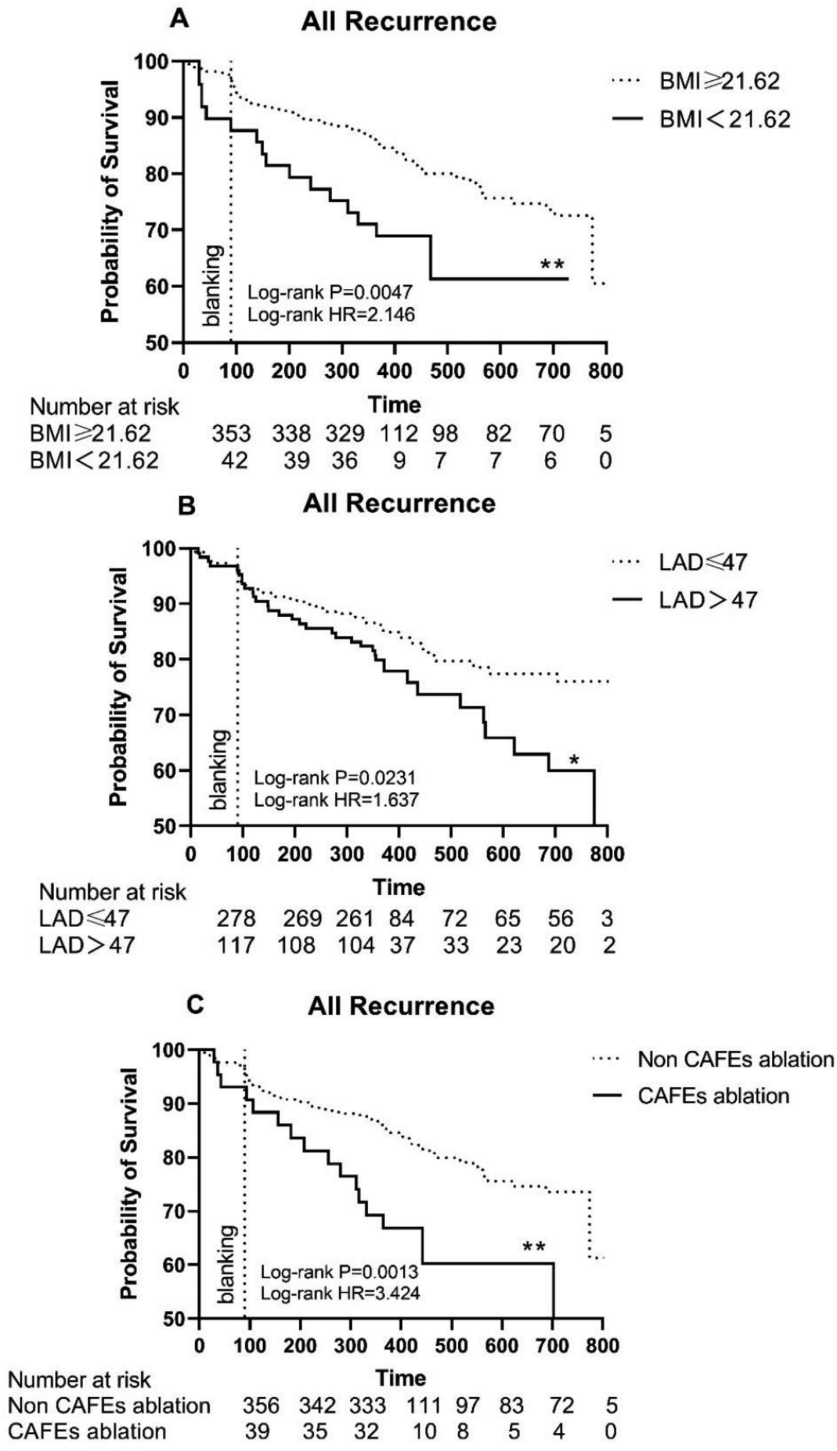
**(A)** The Kaplan–Meier graph showing cumulative survival comparison according to whether BMI ≥ 21.62; **(B)** The Kaplan–Meier graph showing cumulative survival comparison according to whether LAD > 47 mm; **(C)** The Kaplan–Meier graph showing cumulative survival comparison according to whether underwent CAFE ablation. Statistically significant differences are indicated as follows: *P < 0.05; **P < 0.01; ***P < 0.001; ****P < 0.0001. BMI, body mass index; LAD, left atrial diameter; CAFEs, complex fractionated atrial electrograms; HR, hazard ratio.

The multivariate Cox regression analysis identified female sex, BMI, LAD, and CAFE ablation as four independent risk factors as predictors for the primary endpoint events (Table 4). Being female was associated with a 1.96 times higher risk of AF recurrence than being male (95% CI: 1.27–3.02, *P* = 0.002). Similarly, patients with a BMI over 21.62 had a 2.05 times higher risk (95% CI: 1.18–3.56, *P* = 0.011), and patients with a LAD greater than 47 mm had a 1.81 times higher risk (95% CI: 1.17–2.81, *P* = 0.007). Notably, patients who underwent CAFE ablation had a higher AF recurrence rate (HR: 2.11, 95% CI: 1.22–3.66, *P* = 0.008). The Kaplan–Meier survival curve showed a significant difference in the primary endpoint in patients with these risk factors (Figures 5A–C).

After PSM, being female remained a significant risk factor for AF recurrence in the univariate and multivariable Cox regression analyses (HR: 2.20, 95% CI: 1.23–3.76, *P* = 0.007) (Table 4). Variables that were not statistically significant are shown in Supplementary Table S2.

### Subgroup analyses and non-linear relationship analysis

**Figure 6:**
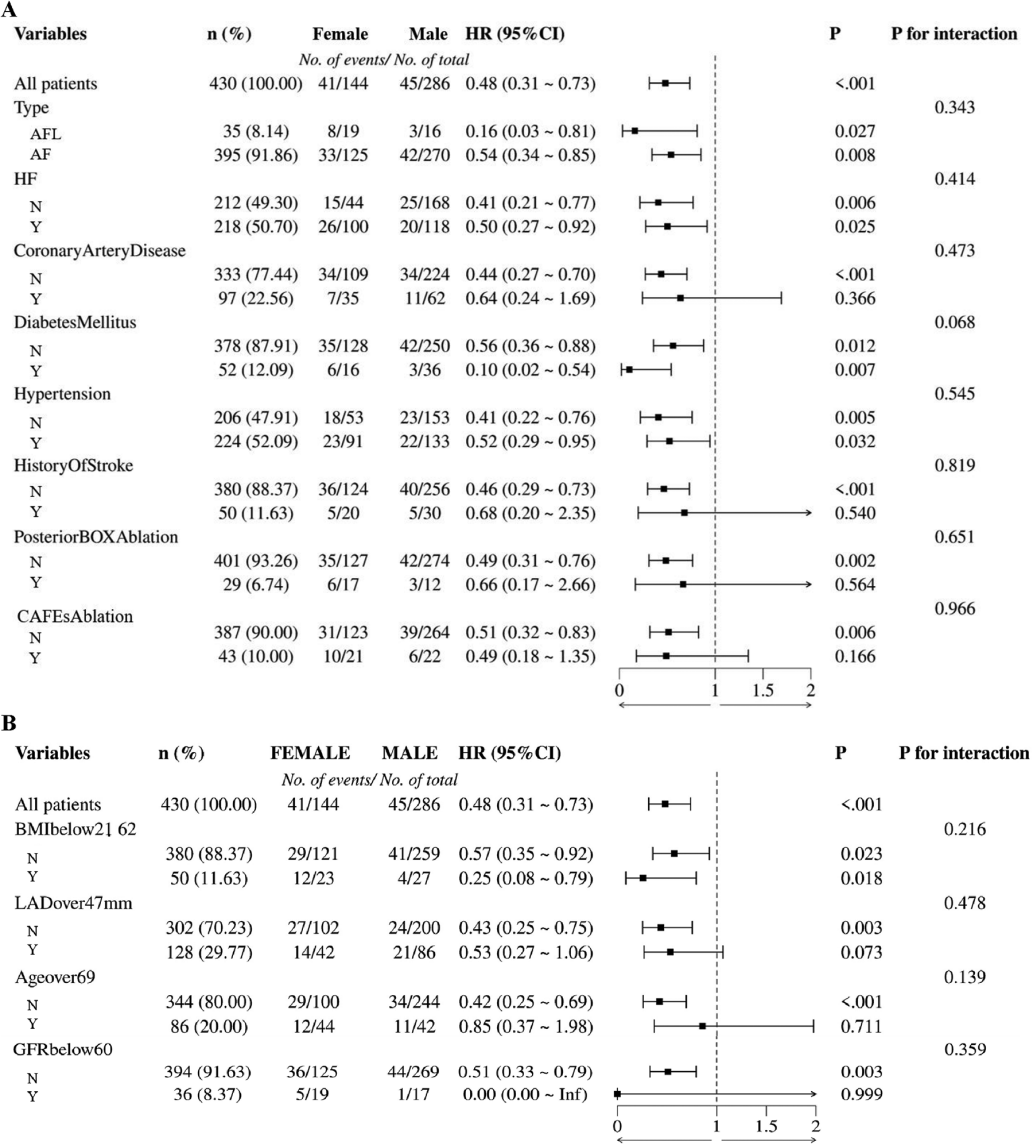
**(A)** Subgroup analysis of first endpoint according to history of disease, ablation site; **(B)** Subgroup analysis of first endpoint according to BMI, age, and others. HR, hazard ratio; CI, confidence interval; AFL, atrial flutter; AF, atrial fibrillation; HF, heart failure; CAFEs, complex fractionated atrial electrograms; BMI, body mass index; LAD, left atrial diameter; GFR, glomerular filtration rate.

To further explore the interactions among the risk factors, we performed subgroup analyses and interaction tests stratified by gender to discover possible population differences. We found no interaction relationship between being female and other factors (all *P* for interaction > 0.05) (Figures 6A,B).

The restricted cubic splines (RCS) analysis of BMI/LAD, adjusted for the effects of LAD/BMI, female sex, and CAFE ablation, suggested an “L-shaped” association of BMI/LAD with the endpoint. The inflection point of the RCS curve was identified at BMI = 25.31/LAD = 45 mm, representing a turning point in the relationship between the BMI/LAD and the endpoint (Supplementary Figure S2).

Using the inflection point, the data were stratified into four groups: BMI <25.31, BMI ≥25.31, LAD <45 mm, and LAD ≥45 mm. Segmented regression was then performed on each group separately, with the results presented in Supplementary Table S3.

The results indicated that BMI showed a non-linear relationship trend (*P* for non-linear = 0.069). In the BMI <25.31 group, the hazard ratio of the endpoint gradually decreased (HR: per SD 0.64, 95% CI: 0.49–0.84, *P* = 0.001), and this trend was not significant in BMI ≥25.31 group (HR: per SD 0.98 95% CI: 0.73–1.32, *P* = 0.88). Similarly, there was no significant non-linear relationship between LAD and the endpoint (*P* for non-linear = 0.516).

## Discussion

The main findings of this study are as follows:

(1) Compared with the male patients, the female patients were older, more symptomatic, had more comorbidities, and were more likely to be complicated with heart failure.
(2) Sex differences in recurrence after EIVOM were significant, and being female was an independent risk factor for AF recurrence.
(3) Low BMI, LAD, and CAFE ablation were other risk factors for recurrence identified in this study.

### Efficacy of EIVOM in women

It is well known that female sex is a risk factor for AF recurrence after catheter ablation (5–8). In a retrospective cohort of patients with persistent AF, being female was associated with a higher risk of AF recurrence after radiofrequency catheter ablation (RFCA) (16). A meta-analysis pooled 19 observational studies (151,370 patients; 34% women) and found that the rate of freedom from AF/AT recurrence was lower in women than men during the follow-up (overall OR 0.75) (5). While recent randomized controlled trials (RCTs) on EIVOM have not confirmed the findings from observational studies (12, 13), this discrepancy may be attributed to variations in the study populations. For example, only approximately 34% of participants in the PROMPT-AF study had a LAD exceeding 45 mm (12), whereas this proportion was significantly higher at 71.5% in our study. Meanwhile, the underrepresentation of women in AF ablation studies has been documented (17), out of 147 RCTs involving 30,055 participants, only 10 trials (6.8%) included women as more than 50% of their participants, and the proportion of female patients in the two RCTs on EIVOM was less than 30% (12, 13). Furthermore, the larger sample sizes in observational studies and meta-analyses of observational studies offer greater statistical power to detect sex-based differences, whereas the smaller and more focused populations in RCTs may lack the necessary statistical power to identify subtle variations in AF recurrence rates.

A higher prevalence of extra-pulmonary vein (PV) triggers of AF in women may be responsible. A study on late recurrence of atrial fibrillation after catheter ablation showed that extra-PV triggers accounted for up to 16.3% of AF recurrence. Furthermore, women who undergo redo AF ablation are more likely to have non-pulmonary vein triggers (18). One study investigated 443 patients with recurred AF after *de novo* ablation. Researchers found that PV reconnections were less prevalent in female patients, while the rate of extra-PV triggers of AF was more common (19). Another study showed that extra-PV triggers were present in up to 28.6% of recurrent AF, and being a woman was independently associated with extra-PV triggers in redo AF ablation (20).

Women tend to have more low-voltage areas compared to men in AF ablation. In a study using high-density electroanatomical maps, women had a fourfold higher risk of having advanced atrial remodeling (low-voltage zone >15%), which was associated with higher AF recurrence after *de novo* ablation (21). Another study also showed a higher prevalence of extensive low-voltage zones in women (21, 22).

The VOM has been implicated as a source of AF triggers, and it contributes to AF maintenance through its modulating electrophysiological properties. EIVOM combined with catheter ablation can improve AF ablation success rates compared to catheter ablation alone (11, 13). EIVOM facilitates the success rate of MI bidirectional block, which plays a crucial role in reducing recurrence after AF ablation (23–25). MB-mediated re-entrant atrial tachycardia accounted for up to 30.2% of the left AT post-AF ablation (26, 27). Moreover, EIVOM may also benefit persistent AF ablation in other aspects, including facilitating left pulmonary vein isolation (28), eliminating non-pulmonary vein triggers (29), and modulating cardiac autonomic nerves (30). However, our findings indicate EIVOM’s inability to enhance AF ablation success in women, suggesting unidentified AF mechanisms remain influential. Unfortunately, detailed electrophysiological mapping outcomes were not available in our study, thus it was not possible to compare the atrial substrate and AF triggers between men and women. Further studies on the LA substrate and AF triggers are needed to elucidate the mechanism of sex differences in AF ablation.

Epicardial adipose tissue may be a candidate factor. One study showed that peri-atrial/total fat ratio was independently predictive of arrhythmia recurrence post-ablation in both sexes, and women had a significantly higher P/T epicardial fat ratio compared to men (31). Another study investigated sex differences in the association between epicardial adipose volume index (EATVI) and left atrial volume index (LAVI) in patients with either sinus rhythm or AF. Researchers found that the relationship between EATVI and LAVI differed between the men and women in the AF groups (32), suggesting EAT’s potential contribution to sex disparities in AF pathology.

Female patients also have different inflammation and fibrosis processes in AF settings. One study supports an association between relaxin-2 and molecules involved in fibrosis, inflammation, and oxidative stress in patients with AF, which are distributed differently in men and women (33).

### Safety of EIVOM in women

In general, EIVOM has demonstrated acceptable safety standards. In a retrospective study investigating the feasibility and complications in over 700 patients receiving EIVOM, a total of 14 serious complications (2.0%) occurred, including 7 cases of tamponade, 4 of stroke, 1 of anaphylactic shock, 1 of atrioventricular block, and 1 of left appendage isolation (34). The VENUS trial reported a severe peri-procedural complication rate, including 1 case of intra-procedural pericardial effusion, 2 of subacute pericardial effusion requiring drainage, 1 of stroke, and 1 of TIA, among 155 patients who underwent EIVOM (13). However, sex subgroup analysis of safety incidents was largely omitted in previous studies. Our study reported similar rates of severe adverse events, and the rate was statistically comparable in the female and male subgroups. The rate of pericardial effusion was high compared to previous studies. This may be due to the diagnostic criteria. We recorded any cardiac effusion visible in pericardial echocardiography. Most pericardial effusions were small (<10 mm) and resolved spontaneously without intervention. The rate of major pericardial effusion requiring drainage was similar to previous studies. The same goes for stroke and TIA. In general, the sex disparities of peri-procedural complications were not significant. Two cases of atrial-esophageal fistula were recorded among the male patients, which was uncommonly high compared to previous studies. However, the difference between the sex subgroups did not reach statistical significance. We found no evidence that EIVOM increases the risk of atrial-esophageal fistula. The two atrial-esophageal cases in our study may have been accidental. However, if tissue near the esophagus is accidentally damaged during EIVOM ablation, it may theoretically increase the risk of atrial-esophageal fistula, which deserves further investigation.

## Conclusion

Compared with the male patients, the female patients were older, more symptomatic, had more comorbidities, and were more likely to be complicated with heart failure. After adjusting for all these differences, sex differences in recurrence after EIVOM remained significant, with being female an independent risk factor.

## Limitation

First, this was a single-center, retrospective, observational study; therefore, the results may be prone to selection bias or information bias. Second, the sample size included in this study was not large enough; therefore, the study was underpowered to detect the safety and efficacy differences between the sex subgroups. Third, recurrence was diagnosed based on symptoms and HOLTER recordings, intra-cardiac loop recording and long-term uni-lead recording were not performed during the follow-up, thus the recurrence rate could be underestimated. Finally, intra-procedural mappings were not available for each patient, therefore comparisons of AF triggers in the LA substrate were not possible in this study.

## Data Availability

The raw data supporting the conclusions of this article will be made available by the authors, without undue reservation.
