# Supplementary material for: Female sex is an independent risk factor for recurrence after ethanol Marshall bundle elimination in atrial fibrillation ablation
Source: Front Cardiovasc Med. 2025 Apr 22;12:1556222. doi: 10.3389/fcvm.2025.1556222 (PMC12052941; doi:10.3389/fcvm.2025.1556222)
Supplement: Supplementary file 1 [file Datasheet1.pdf]

# Supplementary Materials

**Propensity score matching analysis Methods**

Male and female were matched in a 1:1.5 ratio using propensity score matching (PSM) to reduce treatment-selection bias and potential confounding. We adjusted for age, heart failure, hypertension, HAS-BLED score, N-terminal B-type natriuretic peptide (NT-proBNP), glomerular filtration rate (GFR) below 60, anticoagulation, echocardiographic measurements, ethanol infusion of the vein of Marshall (EIVOM) success and ablation strategy which were possibly related to AF recurrence. We calculated a propensity score of 0.1 for maximum execution performance and fixed caliper width. Factors associated with gender were analyzed using logistic regression analyses.

Data processing and analysis were performed using R software (version 4.2.2, R Foundation for Statistical Computing, Vienna, Austria), along with the use of MSTATA software (https://www.mstata.com/).

**Nonlinear relationship analysis Methods**

To explore the nonlinear association between BMI, LAD and Survival using restricted cubic splines (RCS), while adjusting for LAD/BMI, female gender, and CAFEs ablation, we conducted a Nonlinear relationship analysis.

In this study, we collected data on the outcome Survival, the continuous predictor variable BMI/LAD, and covariates LAD/BMI, female gender, and CAFEs ablation. Possible nonlinear relationships between the change in BMI/LAD and Survival were examined by a cox regression model with RCS. The knots between 3 and 7 were tested respectively, and the model with lowest Akaike information criterion value was selected for RCS. Finally, we used RCS with 3 knots at the 10th, 50th and 90th percentiles.

When interpreting the results of an RCS analysis, the 50th percentile value of the predictor variable was chosen as the reference value. The inflection point represented a turning point or boundary between different patterns of association between the predictor variable and the outcome. In cases where the RCS analysis revealed a U-shaped, Inverted U-shaped, or L-shaped curve, with a clearly identifiable inflection point, the data were divided into two distinct segments based on this inflection point. This segmented cox regression allowed for a more nuanced understanding of the relationship between the predictor variable and the outcome in each segment, as it accounted for the distinct patterns of association in different parts of the curve.

The RCS statistical analyses were performed using R software (version 4.2.2, R Foundation for Statistical Computing, Vienna, Austria), along with the use of MSTATA software (https://www.mstata.com/).

**Secondary ablation details**

This was the case in several typical blanking patients undergoing secondary ablation:

The first patient is a male, who experienced chest tightness, diaphoresis, dizziness, and transient amaurosis more than 1 month following the initial ablation procedure. His symptoms were persistent and refractory to AADs. A repeat ablation was conducted 66 days after the initial procedure. During this intervention, PV potentials did not recover. Conduction was restored along the mitral isthmus (MI) line, and additional ablation was performed within the coronary sinus (CS). Simultaneously, CAFEs ablation was also performed.

The second patient is also a male, who developed chest tightness, and dizziness over 1 month after the first ablation. Repeat ablation was performed 74 days after the initial ablation. PV potentials remained absent. 3D Mapping showed that he had mitral-dependent AFL, with conduction was re-established at the MI line. The VOM was absent upon CS angiography. Ablation within LA and CS failed to block the MI line, and the blockage of the MI line was finally achieved via epicardial ablation in the ridge area.

The third patient is a female, who presented with palpitations accompanied by chest tightness and dyspnea more than one month after the initial ablation. ECG revealed AFL. A second ablation was performed 54 days after the first procedure, during which tricuspid isthmus-dependent AFL was identified and ablated.

These 3 patients received their second ablation about 2 months after the first procedure, which is near to the end of the blanking period. They had severe symptoms that were refractory to AADs, therefore with high possibility of secondary ablation even after the blanking period. Thus, we included these patients in the analysis of end-point events.

**TABLE S1**

Cox univariate regression supplementary table before matching

| Variables | HR (95% CI) | P |
|---|---|---|
| **Female gender** | **2.09(1.36,3.21)** | **＜0.001*** |
| Age | 1.01(0.99,1.04) | 0.266 |
| **Age＞69** | **1.60(0.98,2.62)** | **0.061** |
| BMI | 0.97(0.91,1.04) | 0.365 |
| **BMI＜21.62** | **2.16(1.25,3.72)** | **0.006**** |
| Persistence AF | 0.72(0.42,1.22) | 0.220 |
| Time from diagnosis to first AF ablation | 1.01(0.99,1.02) | 0.371 |
| **Time from diagnosis to first AF ablation＞12.0 months** | **1.44(0.94,2.20)** | **0.096** |
| Redo AF/AFL | 1.35(0.70,2.61) | 0.376 |
| Hypertension | 0.94(0.62,1.44) | 0.790 |
| Diabetes | 0.84(0.42,1.68) | 0.618 |
| History of cardiomyopathy | 0.94(0.52,1.84) | 0.935 |
| **Valve replacement** | **2.51(1.09,5.76)** | **0.030*** |
| Coronary artery disease | 0.89(0.53,1.52) | 0.676 |
| CHA2DS2-VASc score | 1.10(0.97,1.24) | 0.128 |
| **CHA2DS2-VASc score ≥5** | **2.00(1.19,3.37)** | **0.009**** |
| HAS-BLED score | 0.93(0.71,1.20) | 0.553 |
| HAS-BLED score ≥2 | 0.77(0.44,1.35) | 0.367 |
| GFR | 0.99(0.98,1.01) | 0.304 |
| GFR＜92 | 1.47(0.91,2.38) | 0.114 |
| LVEF | 0.99(0.97,1.03) | 0.805 |
| **LVEF＜54%** | **1.48(0.97,2.27)** | **0.071** |
| LAD | 1.03(0.98,1.07) | 0.222 |
| **LAD＞47mm** | **1.64(1.07,2.52)** | **0.025*** |
| LVEDD | 0.97(0.93,1.01) | 0.165 |
| **LVEDD＜45mm** | **1.87(1.15,3.04)** | **0.012*** |
| RAD | 1.00(0.97,1.04) | 0.838 |
| **RAD＞40mm** | **1.48(0.94,2.31)** | **0.088*** |
| EIVOM successful | 1.26(0.55,2.90) | 0.580 |
| Anterior linear ablation | 1.46(0.46,4.63) | 0.519 |
| **Posterior BOX ablation** | **2.16(1.08,4.33)** | **0.030*** |
| **CAFEs ablation** | **2.39(1.38,4.12)** | **0.002**** |

Statistically significant differences are indicated as *P <0.05, **P <0.01. Bold values indicate statistically significant results deserving special attention.

Abbreviations: HR=Hazard ratio; CI=Confidence interval; BMI=Body mass index; AF=Atrial fibrillation; AFL=Atrial flutter; GFR=Glomerular filtration rate; SCr= Serum creatinine; LVEF=Left ventricular ejection fraction; LAD=Left atrial diameter; LVEDD=Left ventricular end diastolic diameter; RAD=Right arial diameter; LVEF=Left ventricular ejection fraction; EIVOM=Ethanol infusion of the vein of Marshall; CAFEs=Complex fractionated atrial electrograms.

**TABLE S2**

Cox univariate regression supplementary table after matching

| Variables | HR (95% CI) | P |
|---|---|---|
| **Female gender** | **2.07(1.19,3.60)** | **0.010*** |
| Age | 1.01(0.97,1.03) | 0.934 |
| Age＞69 | 1.40(0.73,2.68) | 0.312 |
| BMI | 1.01(0.92,1.09) | 0.998 |
| **BMI＜21.62** | **1.86(0.93,3.72)** | **0.080*** |
| Persistence AF | 1.08(0.49,2.40) | 0.845 |
| Time from diagnosis to first AF ablation | 1.01(0.99,1.02) | 0.301 |
| Time from diagnosis to first AF ablation＞12.0 months | 1.53(0.91,2.58) | 0.109 |
| Redo AF/AFL | 0.95(0.38,2.37) | 0.908 |
| Hypertension | 0.91(0.52,1.57) | 0.730 |
| Diabetes | 1.18(0.75,1.88) | 0.474 |
| History of cardiomyopathy | 0.84(0.36,1.97) | 0.685 |
| **Valve replacement** | **4.68(1.86,11.82)** | **0.001**** |
| Coronary artery disease | 1.14(0.60,2.16) | 0.692 |
| CHA2DS2-VASc score | 1.03(0.88,1.21) | 0.732 |
| CHA2DS2-VASc score ≥5 | 1.82(0.89,3.75) | 0.103 |
| HAS-BLED score | 0.78(0.55,1.10) | 0.155 |
| **HAS-BLED score ≥2** | **0.48(0.21,1.09)** | **0.078** |
| GFR | 0.99(0.98,1.02) | 0.743 |
| GFR＜92 | 1.71(0.88,3.35) | 0.115 |
| LVEF | 0.99(0.96,1.04) | 0.927 |
| LVEF＜54% | 1.32(0.75,2.30) | 0.336 |
| LAD | 1.04(0.98,1.10) | 0.241 |
| **LAD＞47mm** | **1.69(0.96,2.96)** | **0.068** |
| LVEDD | 0.95(0.90,1.01) | 0.121 |
| LVEDD＜45mm | 1.53(0.81,2.87) | 0.191 |
| RAD | 0.99(0.95,1.04) | 0.736 |
| RAD＞40mm | 1.25(0.71,2.19) | 0.444 |
| EIVOM successful | 2.26(0.55,9.30) | 0.258 |
| Anterior linear ablation | 1.36(0.19,9.89) | 0.761 |
| **Posterior BOX ablation** | **2.53(1.07,5.98)** | **0.035*** |
| **CAFEs ablation** | **1.89(0.95,3.78)** | **0.072** |

Statistically significant differences are indicated as *P <0.05, **P <0.01. Bold values indicate statistically significant results deserving special attention.

Abbreviations: HR=Hazard ratio; CI=Confidence interval; BMI=Body mass index; AF=Atrial fibrillation; AFL=Atrial flutter; GFR=Glomerular filtration rate; LVEF=Left ventricular ejection fraction; LAD=Left atrial diameter; LVEDD=Left ventricular end diastolic diameter; RAD=Right arial diameter; LVEF=Left ventricular ejection fraction; EIVOM=Ethanol infusion of the vein of Marshall; CAFEs=Complex fractionated atrial electrograms.

**TABLE S3**

**A** Effect of BMI Level on Survival: Adjusted Hazard Ratios from Segmented Cox Regression Analysis

| Characteristic | HR | 95% CI | P |
|---|---|---|---|
| **BMI (< 25.31)** | **0.80** | **0.70, 0.92** | **0.001**** |
| BMI (≥ 25.31) | 0.99 | 0.87, 1.13 | 0.88 |

**B** Effect of Standardized BMI Level on Survival: Adjusted Hazard Ratios from Segmented Cox Regression Analysis

| Characteristic | HR per SD | 95% CI | P |
|---|---|---|---|
| **BMI (<25.31)** | **0.64** | **0.49, 0.84** | **0.001**** |
| BMI (≥25.31) | 0.98 | 0.73, 1.32 | 0.88 |

**C** Effect of LAD Level on Survival: Adjusted Hazard Ratios from Segmented Cox Regression Analysis

| Characteristic | HR | 95% CI | P |
|---|---|---|---|
| LAD (<45mm) | 1.02 | 0.92, 1.13 | 0.74 |
| LAD (≥45mm) | 1.05 | 0.95, 1.16 | 0.34 |

**D** Effect of Standardized LAD Level on Survival: Adjusted Hazard Ratios from Segmented Cox Regression Analysis

| Characteristic | HR per SD | 95% CI | P |
|---|---|---|---|
| LAD (<45mm) | 1.06 | 0.77, 1.46 | 0.74 |
| LAD (≥45mm) | 1.15 | 0.87, 1.51 | 0.34 |

Statistically significant differences are indicated as *P <0.05, **P <0.01.

Abbreviation: HR = Hazard ratio; SD= Standard deviation; CI = Confidence interval; BMI=Body mass index; LAD=Left atrial diameter; CAFEs=Complex fractionated atrial electrograms

HRs were adjusted for BMI, gender, and CAFEs ablation.

**FIGURE S1**

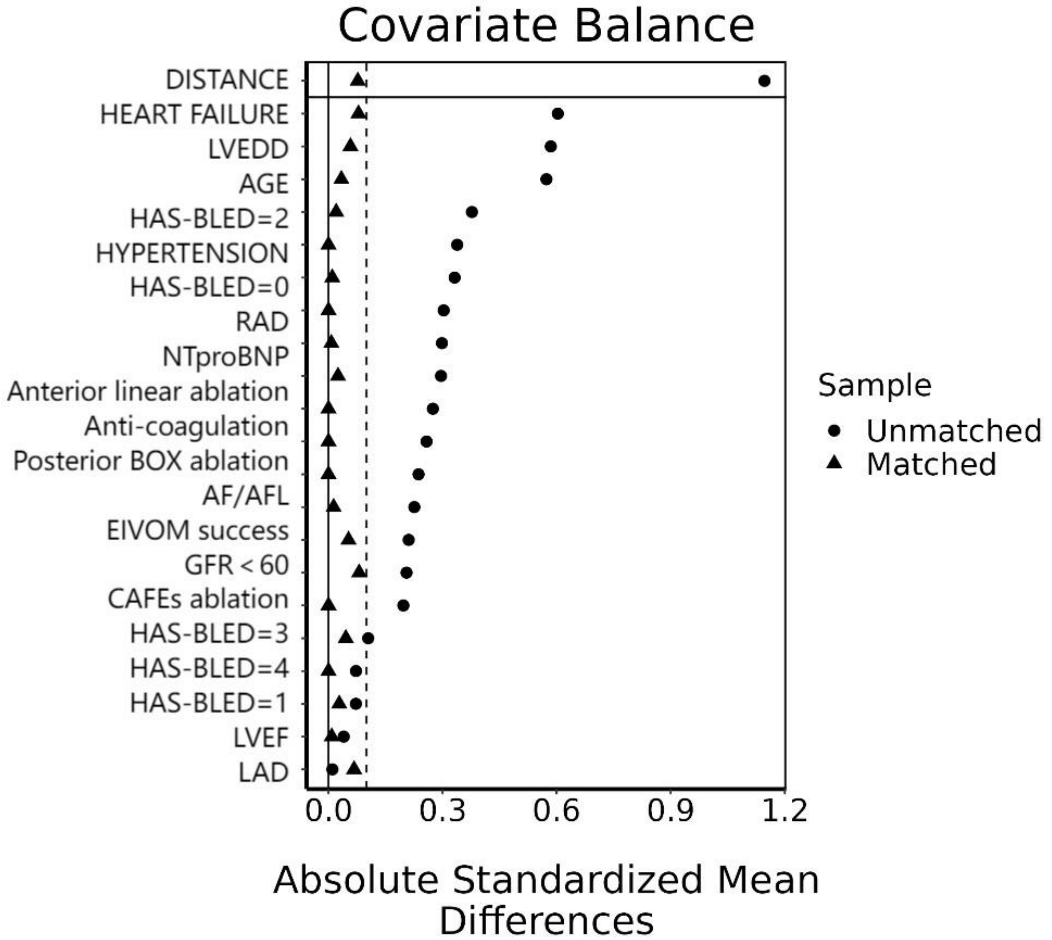


A scatter plot that displays the absolute standardized mean differences (SMD) of various covariates before and after propensity score matching.

Abbreviations: AF=Atrial fibrillation; AFL=Atrial flutter; LVEDD=Left ventricular end diastolic diameter; LVEF=Left ventricular ejection fraction; LAD=Left atrial diameter; RAD=Right arial diameter; LVEF=Left ventricular ejection fraction; EIVOM=Ethanol infusion of the vein of Marshall; CAFEs=Complex fractionated atrial electrograms; GFR=Glomerular filtration rate; NT-ProBNP=N-terminal B-type natriuretic peptide.

**FIGURE S2**

**A**

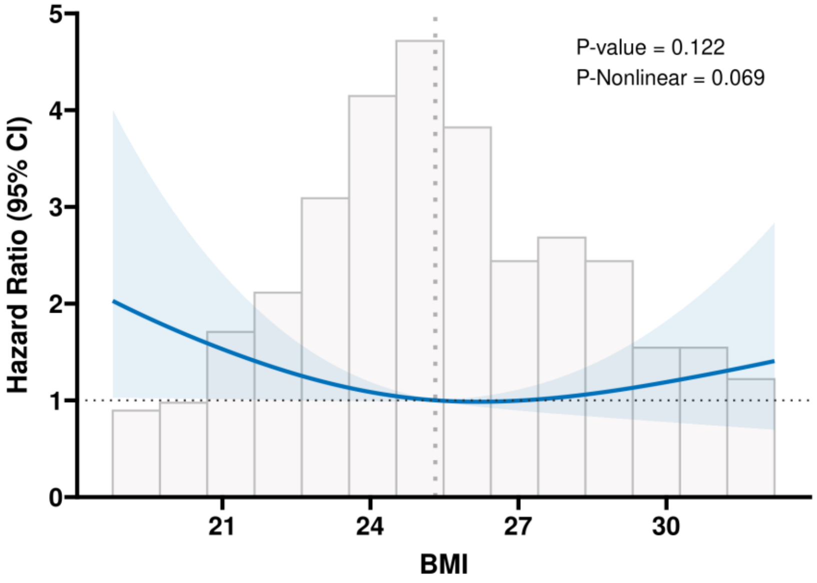


**B**

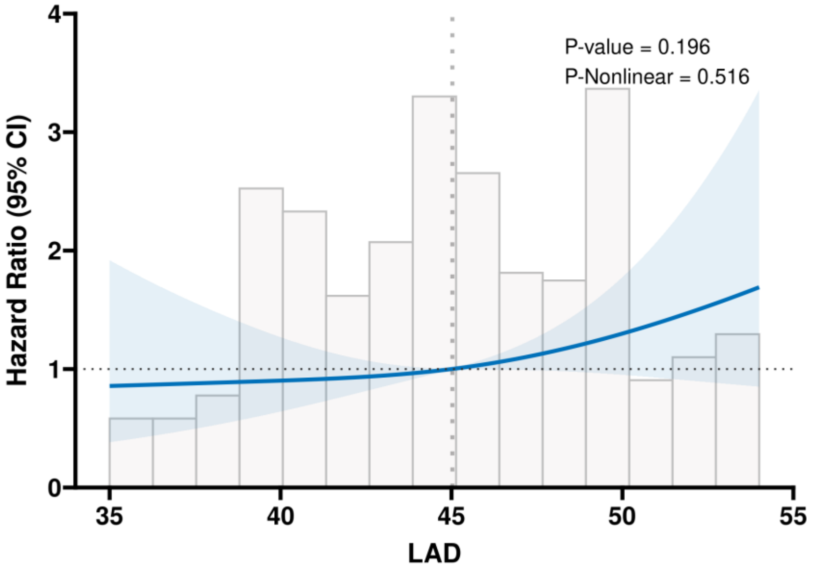


Abbreviation: RCS= Restricted cubic spline; BMI=Body mass index; LAD=Left atrial diameter; CAFEs=Complex fractionated atrial electrograms; HR=Hazard ratio;

A: Association between BMI and Survival with the RCS function. Model with 3 knots located at 10th, 50th and 90th percentiles. Y-axis represents the HR to present survival at the primary endpoint for any value of BMI compared to individuals with reference value (50th percentile) of BMI. The Cox regression was adjusted for LAD, gender, and CAFEs ablation.

B: Association between LAD and Survival with the RCS function. Model with 3 knots located at 10th, 50th and 90th percentiles. Y-axis represents the HR to present survival at the primary endpoint for any value of LAD compared to individuals with reference value (50th percentile) of LAD. The Cox regression was adjusted for BMI, gender, and CAFEs ablation.
